# New Insights into the Dissolution Kinetics of Alite Powder and the Effects of Organic Toughening Materials

**DOI:** 10.3390/ma16227242

**Published:** 2023-11-20

**Authors:** Jinhui Tang, Guangye Tu, Zongshuo Tao, Yu Yan

**Affiliations:** 1 School of Materials Science and Engineering, Jiangsu Key Laboratory of Construction Materials, Southeast University, Nanjing 211189, China; 101012824@seu.edu.cn (J.T.); 220232655@seu.edu.cn (G.T.); 2 China Building Materials Test and Certification Group Anhui Co., Ltd., Hefei 230051, China; coonsu@163.com; 3 Department of Civil and Environmental Engineering, National University of Singapore, Singapore 117576, Singapore

**Keywords:** alite, powder, dissolution, organic toughening materials

## Abstract

Alite dissolution plays a crucial role in cement hydration. However, quantitative investigations into alite powder dissolution are limited, especially regarding the influence of chemical admixtures. This study investigates the impact of particle size, temperature, saturation level, and mixing speed on alite powder dissolution rate, considering the real-time evolution of specific surface area during the alite powder dissolution process. Furthermore, the study delves into the influence of two organic toughening agents, chitosan oligosaccharide (COS) and anionic/non-ionic polyester-based polyurethane (PU), on the kinetics of alite powder dissolution. The results demonstrate a specific-surface-area change formula during alite powder dissolution: $\frac{S}{S_{0}}=0.348e^{\left(1-m/m_{0}\right)/0.085}+0.651$. Notably, the temperature and saturation level significantly affect dissolution rates, whereas the effect of particle size is more complicated. COS shows dosage-dependent effects on alite dissolution, acting through both its acidic nature and surface coverage. On the other hand, PU inhibits alite dissolution by blocking the active sites of alite through electrostatic adsorption, which is particularly evident at high temperatures.

## 1. Introduction

During the early age of cement hydration, a dominant role is played by C_3_S (tricalcium silicate, or alite with impurities inside), forming primary hydration products, e.g., calcium silicate hydrate (C-S-H) gel and portlandite (CH), to fill the gap between adjacent cement particles and contributing to the solidification of cement-based materials [1,2,3]. The dissolution behavior of C_3_S, as the very first step of C_3_S hydration, significantly impacts the formation of subsequent hydration products and the development of the microstructure, which, in turn, governs the macroscopic properties of the cementitious matrix [4]. However, despite its crucial influence, a comprehensive understanding of the mechanisms driving its dissolution process remains a challenge, and addressing this is essential for improving our understanding of cement hydration and for optimizing concrete design.

Dissolution theory in geochemistry has made significant advancements in comprehending the dissolution patterns of crystals [5,6]. The use of advanced testing techniques, such as digital holographic microscopy (DHM), vertical scanning interferometry (VSI), and atomic force microscopy (AFM), has enabled a more comprehensive quantitative analysis of the dissolution kinetics of single-crystal minerals [7,8,9]. Note that the dissolution behavior of powdered minerals significantly deviates from that of bulk crystals. Typically, the rates observed during the dissolution of single crystals are 10 to 1000 times lower than those recorded in stirred suspensions of powders [9,10].

Expanding upon the dissolution theory, Patrick et al. [11,12,13] and Nicoleau et al. [4,14,15] established and refined the dissolution models suitable for C_3_S/alite hydration. The dissolution reaction of C_3_S occurs as follows [16]:

(1) $${Ca}_{3}SiO_{5}+5H_{2}O\to 3{Ca}^{2+}+6{OH}^{-}+H_{4}SiO_{4}$$

In the initial stage of C_3_S dissolution (after contacting water), etch pits are formed on its surface, accompanied by a rapid release of ions into the pore solution and significant heat release. As ion concentrations gradually rise in the pore solution, the degree of undersaturation with regard to C_3_S decreases, resulting in a notable slowdown in the dissolution rate. When the driving force is no longer sufficient to overcome the activation energy required to create new etch pits, a slow dissolution mechanism, known as step-retreat dissolution occurring under nearly equilibrium conditions, takes control [11,17]. The critical role of solution saturation levels in the C_3_S dissolution process can be further revealed by the comparison of the surface microstructure of alite powder dissolved in pure water versus saturated lime solution. In pure water, extensive surface pitting occurs within just a few minutes, whereas in a saturated lime solution, most of the surface remains smooth and unattacked, even after 30 min [11]. Beyond the saturation levels, the presence of different ions in the pore solution can also influence the C_3_S dissolution process. For example, studies by Nicoleau et al. [15] have shown that monovalent cations and anions have minimal interactions with the surface of C_3_S. However, divalent anions like sulfate significantly slow down dissolution by modifying the surface charge of C_3_S, while aluminate ions covalently bind to surface silicate monomers, inhibiting dissolution in mildly alkaline conditions. Furthermore, factors such as crystal orientation, defect density, temperature, particle size distribution (PSD), and mixing rate all play roles in the dissolution of C_3_S. Consequently, these factors can alter the onset time of the induction period and the ongoing hydration of C_3_S [18,19,20]. As reported in the existing literature, the dissolution rates of C_3_S exhibited a wide range, spanning from less than 1 μmol·m^−2^·s^−1^ to over 100 μmol·m^−2^·s^−1^, depending on the characteristics of C_3_S and the specific pore-solution environment [13,21].

With the demand for high-performance and low-carbon concrete, except for the use of supplementary cementitious materials (SCMs) [22,23] and recycled materials [24], adding chemical admixtures has been a common and effective technique in modern concrete preparation [25,26,27,28]. The application of organic biomaterials to influence the cement hydration process and systematically regulate the morphology of hydration products has emerged as a prominent research area in achieving the biomineralization and toughening of cement-based materials [29]. Research findings demonstrate that the incorporation of organic molecules, e.g., polyurethane [30], chitosan [31], ethylene vinyl acetate copolymer (EVA) [32], and polyvinyl alcohol (PVA) [33], can create an interconnected network between organic and inorganic components in the hydration products, leading to an improvement in the mechanical properties, particularly in the toughness of cement-based materials. Considering the distinct physical and chemical properties of organic toughening materials and their potential perturbation of the intricate cement hydration kinetics, it is imperative to conduct more quantitative investigations into how organic molecules impact the dissolution process of C_3_S/alite.

In this study, we investigated the dissolution behaviors of alite powder, considering the change in specific surface area by various influencing factors, including particle size distribution, mixing speed, temperature, and saturation level of the pore solution. On this basis, the effects of COS and PU, two well-known organic toughening materials, on the dissolution kinetics of alite powder were further studied.

## 2. Materials and Methods

### 2.1. Materials

**Figure 1:**
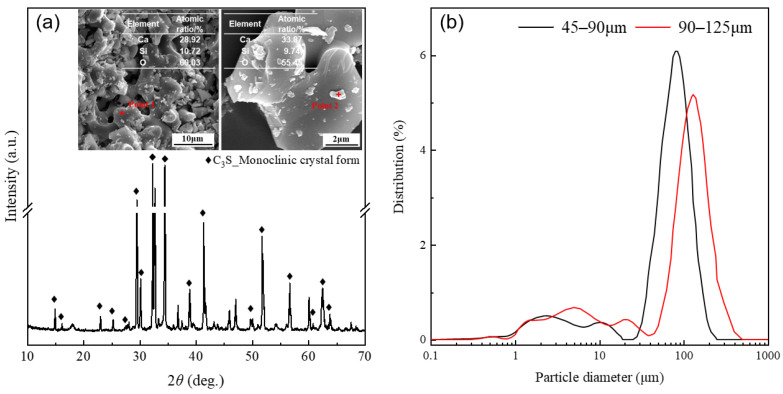
(**a**) XRD pattern and typical SEM images of the synthesized alite powder. (**b**) Apowder with two different particle size distributions: 45–90 μm and 90–125 μm.

The alite used in this study was self-synthesized in our laboratory according to the method of X. Li et al. [34]. Initially, accurately weigh calcium carbonate CaCO_3_ and silica SiO_2_ (4N, Sinopharm Chemical Reagent Co., Ltd., Shanghai, China) in a 3:1 molar ratio. A small amount of Al_2_O_3_, MgO, and Fe_2_O_3_ (4N, Sinopharm Chemical Reagent Co., Ltd.) was added to promote grain growth, stabilize the monoclinic structure of the alite, and to mimic the alite found in Portland cement. Additionally, a mineralizer, CaF_2_, was included. The mixture was thoroughly blended using a mixing machine. Subsequently, the mixed powder was sintered at 1500 °C for 8 h and quenched in air. This process was repeated three times to improve the purity of the synthesized alite. The chemical composition of the resulting alite can be found in Table 1. Finally, the chunks of alite were ground and sieved into two batches of alite powders differing in particle size distribution: 45–90 μm and 90–125 μm, as determined by a particle size analyzer PSA1190LD from Anton Paar (North Ryde, Australia). The X-ray diffraction (XRD) pattern (measured by a Bruker D8 Advance diffractometer with a CuKα source at a scanning rate of 0.02°/s) and particle size distribution of the alite powders used for the dissolution measurements are presented in Figure 1.

The effect of two organic toughening agents, chitosan oligosaccharide (COS), and anionic/non-ionic polyester-based polyurethane (PU), on the dissolution kinetics of alite powder was also studied. COS, with the chemical formula (C_12_H_24_N_2_O_9_)_n_, was procured from Shanghai Aladdin Bio-Chem Technology Co., Ltd. (Shanghai, China). It appears as a light-yellow solid powder and exhibits high solubility due to its relatively low molecular weight (typically ≤2000 Da) and its molecular structure with amino and hydroxyl groups. Anionic/non-ionic polyester polyurethane (Baybond^®^ PU 401 A) was procured from Covestro AG (Leverkusen, Germany). This material is supplied in the form of a milky white dispersion, readily dilutable in water at any desired ratio. To adjust the pore solution’s saturation level, we utilized calcium hydroxide acquired from Sinopharm Chemical Reagent Co., Ltd. Ultrapure water (UPW, 18.2 MΩ·cm) was used throughout our experiments.

### 2.2. Methods

**Figure 2:**
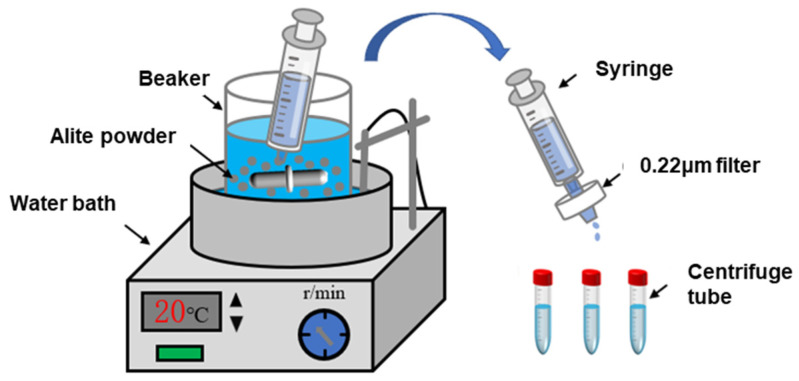
Diagram of the alite powder dissolution test device.

Figure 2 illustrates the reaction setup used for the dissolution of the alite powder. The reaction vessel is a custom-made plastic container equipped with a magnetic stirring rotor. In each dissolution experiment, 500 mL of liquid (ultrapure water or a pre-made solution) was added to the container and placed in a water bath to maintain a controlled temperature of 20 ± 1 °C or 40 ± 1 °C. Once the solution temperature had stabilized, 0.5 g of alite powder was introduced into the container, maintaining a liquid-to-solid ratio of 1000 (to prevent precipitation of hydration products, as per Nicoleau et al. [4]). After a specified time, approximately 5 mL of the suspension was withdrawn using a syringe, and the extracted liquid was rapidly filtered through 0.22 μm nylon syringe filters for immediate separation. The duration from alite powder addition to completion of the filtration was approximately 10 s. After the filtration, 0.5 mL of 5% nitric acid solution was immediately added to the sample to stabilize the dissolved components in the solution. The solution was then sealed in centrifuge tubes and stored in a refrigerator at 4 °C for further analysis. Finally, the calcium-ion concentration in the samples was measured using inductively coupled plasma–optical emission spectroscopy (ICP-OES). The ion activities and supersaturation level for alite dissolution were calculated with the Davies equation and the extended Debye–Hückel approach using the PHREEQC 3.7.3 software.

The specific surface area of the alite powder before and after dissolution was measured by N_2_ adsorption–desorption measurements (Micromeritics Tristar II 3020, Norcross, GA, USA) at 77.35 K. Prior to the analysis, the samples underwent degassing within an external degassing station (Micromeritics VacPrep 061, Norcross, GA, USA) at a temperature of 40 °C and under a continuous flow of nitrogen for a duration of 16 h. The specific surface area of the alite powder was calculated by the BET (Brunauer, Emmett, Teller) method.

Scanning electron microscopy (Quanta 3D FEG, FEI, Hillsboro, OR, USA) was used to observe the surface morphology of the alite powder before and after dissolution. Upon reaching the specific time, the alite powder was centrifuged from the suspension and immersed in excess isopropanol to stop hydration. Then, the isopropanol was removed, and the alite powder was dried in a desiccator (45 °C) for approximately 24 h. Finally, the dried alite powder was evenly dispersed onto an adhesive carbon tab and coated with gold before SEM observation. The surface morphology of the dissolved alite powder was observed using the secondary electron mode, and energy-dispersive spectrometry (EDS) was utilized to perform point analysis on the chemical composition of selected areas. The acceleration voltage was fixed at 20 kV, and the working distance was around 8.5 mm.

## 3. Results and Discussion

### 3.1. Dissolution Kinetics of Alite Powder

In accordance with the definition of the dissolution rate, the molar amount of alite consumed per unit time and unit surface area, denoted as μmol·m^−2^·s^−1^, is calculated using the formula in Equation (2):

(2) $$J=-\frac{1}{A}\frac{dN}{dt}=\frac{V}{mS}\frac{dc}{dt}$$

Here, *J* (μmol·m^−2^·s^−1^) represents the dissolution rate, *A* (m^2^) is the total surface area of the alite powder, *N* (mmol) is the amount of dissolved alite powder, *t* (s) is the dissolution time, *V* (m^3^) is volume of the solution, *m* (g) represents the mass of the alite powder, *S* (g·m^−2^) represents the specific surface area of the alite powder, and *c* (mmol·m^−3^) is the ion concentration in the pore solution.

In the actual dissolution process, both the specific surface area and the mass of the powder continuously change as the dissolution progresses. The change in powder mass directly correlates with the ion concentration change in the pore solution, and can be expressed by Equation (3):

(3) $$1-\frac{m\left(t\right)}{m_{0}}=\frac{VM}{m_{0}}[c(t)-c_{0}]$$

The change in specific surface area over the dissolution process has yet to be quantitatively reported. In most cases, they have been neglected [4] or calculated through geometric estimations.

**Figure 3:**
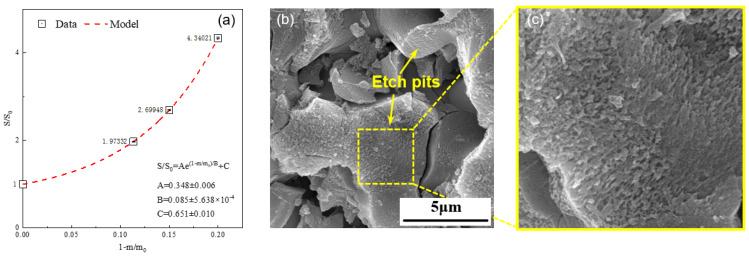
(**a**) Change in specific surface area of the alite powder (90–125 μm) during its dissolution process in ultrapure water. (**b**,**c**) SEM image of the alite powder dissolved in ultrapure water for 150 s.

Figure 3a illustrates the variation in the specific surface area of the alite powder (90–125 μm) during the dissolution process. It can be clearly seen that, with the progression of dissolution, the specific surface area of the alite powder gradually increases. Quantitatively speaking, with 12% of alite powder dissolved (60 s after mixing), the specific surface area increases by over 100%. Further increasing the dissolution degree to 20% (240 s after mixing), the specific surface area of the dissolved alite is more than four times that of the original powder. The microscopic morphology of the alite powder after 150 s of dissolution (Figure 3b,c) validates this trend. In detail, during the dissolution process, the surface of alite powder forms numerous etch pits, presenting a scale-like structure. This phenomenon significantly contributes to the enlargement of the specific surface area of the alite powder. It is noteworthy that no hydration products can be observed on the overall surface of alite particles, indicating that the alite powder undergoes a pure dissolution process without the precipitation of hydration products.

The change in specific surface area of the alite powder was determined through a nonlinear fitting approach, as shown in Equation (4), with the corresponding coefficients *A*, *B*, and *C* shown in Figure 3a. In the rest of this study, we assume that the change in specific surface area of the alite powder during the dissolution process follows a consistent pattern:

(4) $$\frac{S}{S_{0}}=Ae^{\left(1-m/m_{0}\right)/B}+C=Ae^{VM(c-c_{0})/m_{0}B}+C$$

The average dissolution rate of the alite powder can be calculated according to Equations (5) and (6), depending on the change in calcium concentration from $c_{0}$ to c.

(5) $$J=\frac{1}{t}\int_{0}^{t}\frac{V}{m\left(\tau \right)S\left(\tau \right)}\frac{dc}{d\tau }d\tau =\frac{1}{t}\int_{c_{0}}^{c}\frac{V}{m\left(c\right)S\left(c\right)}dc$$

with $x=\frac{VM}{m_{0}}\left(c-c_{0}\right)$, Equation (5) can then be written as follows:

(6) $$J=\frac{1}{S_{0}Mt}\int_{0}^{VM(c-c_{0})∕m_{0}}\frac{dx}{\left(1-x\right)\left(Ae^{x/B}+C\right)}$$

In the following experiments, we measured the change in calcium concentration during a dissolution period of 10 ± 1 s. Note that, in the dissolution reaction equation for alite, the stoichiometric ratio of Ca^2+^ and C_3_S is 3:1. This ratio should be taken into account when calculating the dissolution rate of the alite powder.

### 3.2. Factors Affecting Alite Powder Dissolution

**Figure 4:**
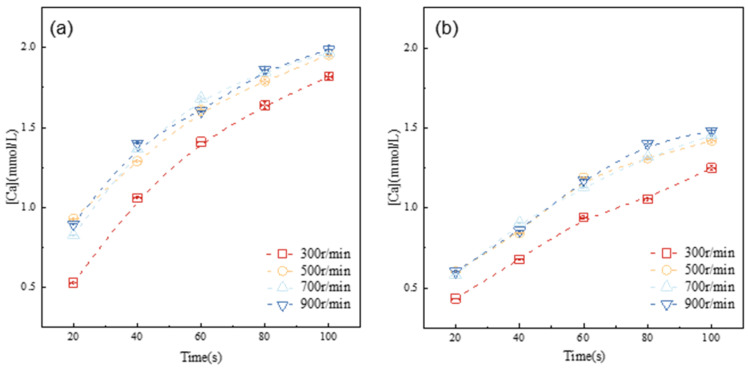
Evolution of calcium concentration in the pore solution of alite suspensions with different particle size distributions at 20 °C: (**a**) 45–90 μm, (**b**) 90–125 μm.

Alite dissolution is characterized by the formation and enlargement of surface etch pits, as well as by the diffusion of ions within the solution [20]. The dissolution rate is primarily controlled by the local ion concentration at the solid–water interface, although experimental measurements generally encompass the overall concentration. When the diffusion rate is slower than the alite dissolution, dissolved ions tend to accumulate on the surface of the anhydrous alite, resulting in a lower dissolution rate. To thoroughly investigate the dissolution kinetics of the alite powder and to mitigate the diffusion-related impact on dissolution rate measurements, experiments were designed to evaluate the effect of varying stirring speeds on the dissolution of the alite powder in ultrapure water, as illustrated in Figure 4.

The results clearly demonstrate that as the stirring speed is elevated from 300 r/min to 500 r/min, there is a noticeable increase in ion concentration within the same dissolution period. However, upon reaching a stirring speed of 500 r/min, the development of ion concentration in the solution shows minimal variation with stirring speed. This suggests that, at this point, dissolution of the alite powder is no longer significantly influenced by ion diffusion. Hence, for the subsequent experiments, the stirring speed was consistently maintained at 500 r/min.

**Figure 5:**
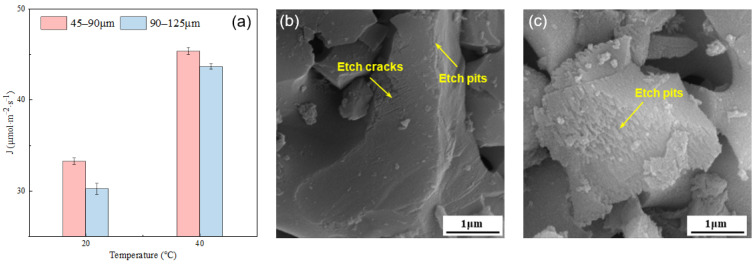
(**a**) Effect of temperature on the dissolution rate of the alite powder with different particle size distributions. SEM image of alite powder with a PSD of 45–90 μm dissolved in ultrapure water for 60 s at (**b**) 20 °C and (**c**) 40 °C.

The influence of temperature on the dissolution rate of the alite powder is presented in Figure 5. For the fine alite powder (PSD 45–90 μm), the dissolution rate at 20 °C is 33.26 μmol·m^−2^·s^−1^, and at 40 °C, it increases to 45.33 μmol·m^−2^·s^−1^, representing a 36.3% increase. Similarly, for alite powder with a PSD of 90–125 μm, the dissolution rate at 20 °C is 30.22 μmol·m^−2^·s^−1^, while at 40 °C, it rises to 44.64 μmol·m^−2^·s^−1^, indicating a 44.41% increase. These results clearly indicate that an increase in temperature significantly enhances the dissolution rate, underscoring the pronounced influence of temperature on dissolution rates of the alite powder.

Figure 5b,c show the surface morphology of alite powder with a particle size range of 45–90 μm after 60 s of dissolution at 20 °C and 40 °C, respectively. At 20 °C, there is slight etching primarily occurring at the particle edges, along with a noticeable etching crack on the surface. This could be attributed to the grinding process that occurred prior to dissolution, which may have introduced lattice defects, thereby facilitating rapid etching during dissolution. In contrast, at 40 °C, extensive etch pits are prominently visible on the alite surface, with pronounced etching occurring at the particle edges. These observations align with the previously mentioned changes in dissolution rates, illustrating the significant impact of temperature on the dissolution process.

**Figure 6:**
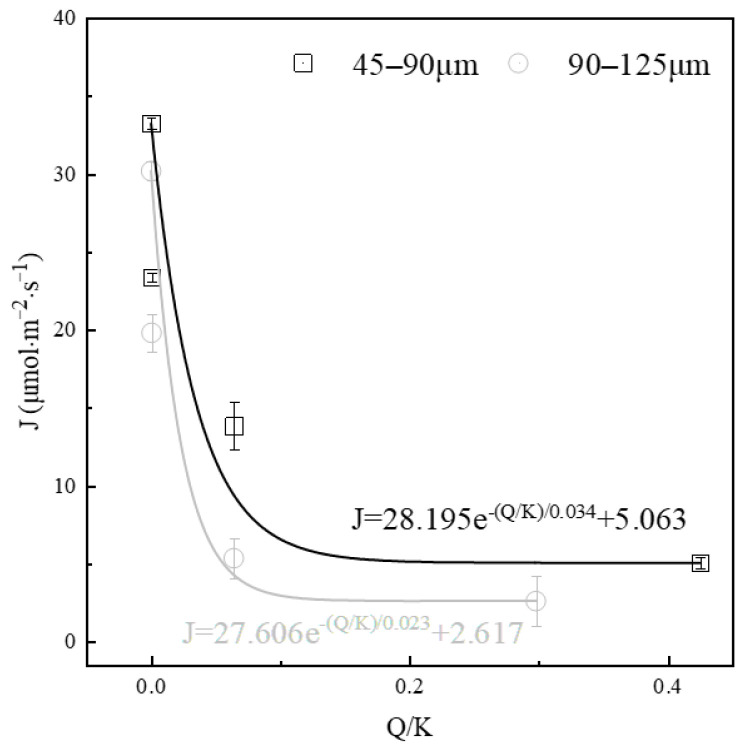
Effect of saturation level on the dissolution rate of alite powder.

The variation in the dissolution rate of alite powder in calcium hydroxide solutions with different degrees of saturation was also evaluated and shown in Figure 6. The saturation degree of calcium hydroxide can be calculated using the equation:

(7) $$\frac{Q}{K}=\frac{\left\{{Ca}^{2+}\right\}{\left\{{OH}^{-}\right\}}^{2}}{K},\;K=5.5\times 10^{-6}$$

The activities of various ions were computed using the thermodynamic software PHREEQC, and the corresponding solution saturation level was calculated, as summarized in Table 2. The results indicate a clear decrease in the dissolution rate of alite powder with an increase in the initial solution saturation. For fine alite powder (45–90 μm), in comparison with a solution saturation level of 0 (dissolving in ultrapure water, where the dissolution driving force is at its maximum), the dissolution rates decrease by 29.71%, 58.36%, and 84.79% with increasing calcium hydroxide concentrations of 0.87 mM, 6.2 mM and 13.4 mM, respectively. An overall trend of exponential decline in the alite powder dissolution rate over the saturation degree of calcium hydroxide solution can be fitted. A similar trend can be observed in the coarse alite powder group (90–125 μm), with a more severe drop in the low saturation stage and a lower stabilized dissolution rate in the high saturation stage.

When considering the effect of particle size distribution on alite powder dissolution, the experimental results mentioned earlier clearly indicate that smaller particle sizes result in higher dissolution rates. However, it is worth noting that the difference in dissolution rates among alite powders of various particle size distributions is relatively minor. As shown in Table 3, in comparison with data from relevant studies, notably Nicoleau et al.’s investigation [4], it becomes apparent that the dissolution rate does not significantly decrease with an increase in particle size. Instead, it is the crystal type of alite that appears to exert a more distinct influence on the dissolution rate. In contrast to the dynamic change in specific surface area during the dissolution process of alite powder, the impact of PSD on alite powder dissolution seems to be more complex. While a finer particle size distribution corresponds to a higher specific surface area, the potential influence of other factors (e.g., defect density, crystal orientation) cannot be ignored. Further in-depth research is needed to better understand the influence of particle size on the dissolution kinetics of alite powder.

### 3.3. Effect of Organic Toughening Materials on the Dissolution of Alite Powder

Blending organic toughening materials with cementitious matrices is one of the pathways to improve the quasi-brittleness characteristic of concrete. The presence of organic toughening materials could severely disturb the dissolution of cement particles and, therefore, change the hydration kinetics of the cement paste. Building upon the investigation of alite powder dissolution kinetics, we further explored the influence of two organic toughening materials, COS and PU, on alite powder dissolution.

**Figure 7:**
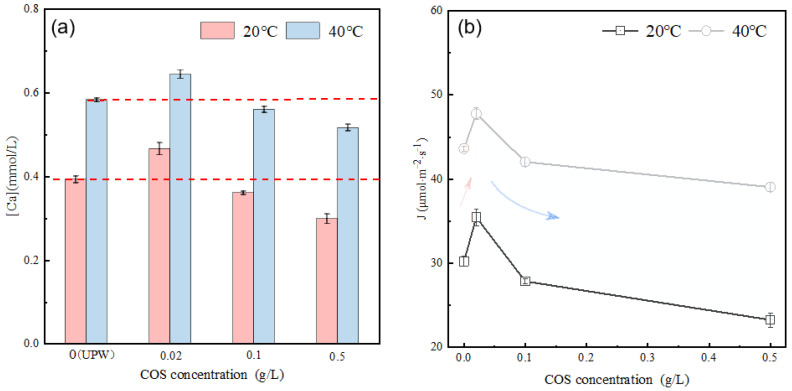
(**a**) Effect of COS concentration on the calcium concentration in the alite (90–125 μm) pore solution after 10 s of dissolution. (**b**) Effect of COS concentration on the dissolution rate of alite powder (90–125 μm).

Figure 7a illustrates the calcium ion concentration in the alite (90–125 μm) suspension with varying COS concentrations after 10 ± 1 s of dissolution. The results demonstrate that, compared with the dissolution in ultrapure water (at both 20 °C and 40 °C), COS affects the dissolved calcium ion concentration, and this effect is directly related to the COS content. Specifically, at low concentration (0.02 g/L), the dissolved calcium ion concentration is higher compared with the pure water condition, showing a temperature-dependent increase of 10–20% temperature-dependent. As the COS concentration increases, the dissolved calcium ion concentration gradually decreases. The average dissolution rate of alite powder in the COS-modified system is further calculated, as shown in Figure 7b. At 20 °C, in comparison with dissolution in ultrapure water (30.22 μmol·m^−2^·s^−1^), at low concentration (0.02 g/L), COS promotes the dissolution of alite powder, resulting in an approximate 17.37% increase in the dissolution rate. However, at higher concentrations (0.1 g/L and 0.5 g/L), it inhibits the dissolution, leading to reductions of about 7.81% and 22.96%, respectively. At 40 °C, the effects are consistent with those at 20 °C, but the overall dissolution rates are faster.

**Figure 8:**
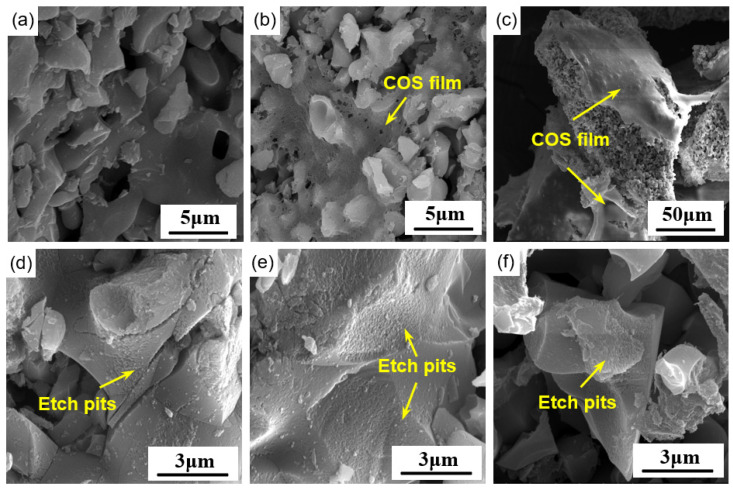
SEM image of dissolved alite powders (90–125 μm) with various COS concentrations at 20 °C: 0.02 g/L COS for (**a**,**d**); 0.10 g/L COS for (**b**,**e**); 0.50 g/L COS for (**c**,**f**). The dissolution time is 60 s.

Figure 8 shows the surface morphology of alite powder with a particle size of 90–125 μm after 60 s of dissolution in COS solutions of different concentrations. The influence of COS on the dissolution of alite powder can be explained from two aspects. Firstly, as the COS concentration increases, varying degrees of COS attachment appear on the particle surfaces. At a concentration of 0.02 g/L, COS attachment is barely observable. As the COS concentration reaches 0.1 g/L, a thin layer of COS becomes noticeable on the alite particle surface. With a further increase in COS concentration to 0.5 g/L, most particle surfaces are covered by COS, and the thickness of the COS layer is significantly greater than the 0.1 g/L condition. Therefore, as the COS concentration increases, the available surface for alite dissolution decreases, leading to a reduction in alite powder dissolution and calcium ion concentration in the solution. Secondly, in all three COS-modified systems, substantial etch pits are observed, exhibiting a step-retreat dissolution pattern independent of COS concentration. In comparison with dissolution in ultrapure water (as shown in Figure 5b), the extent of surface etching on the alite particles is significantly greater, which is likely due to the acidic nature of the COS solution. The effects of these two factors are directly related to the COS concentration: At low concentrations, the acidic properties of COS dominate, resulting in a promoting effect on dissolution. Conversely, at high concentrations, the surface coverage effect of COS dominates, leading to an inhibitory effect on dissolution. Moreover, higher concentrations could result in a greater complexation effect on cations (mainly calcium ions) in the pore solution, which further disturbs the dissolution of the alite powder.

**Figure 9:**
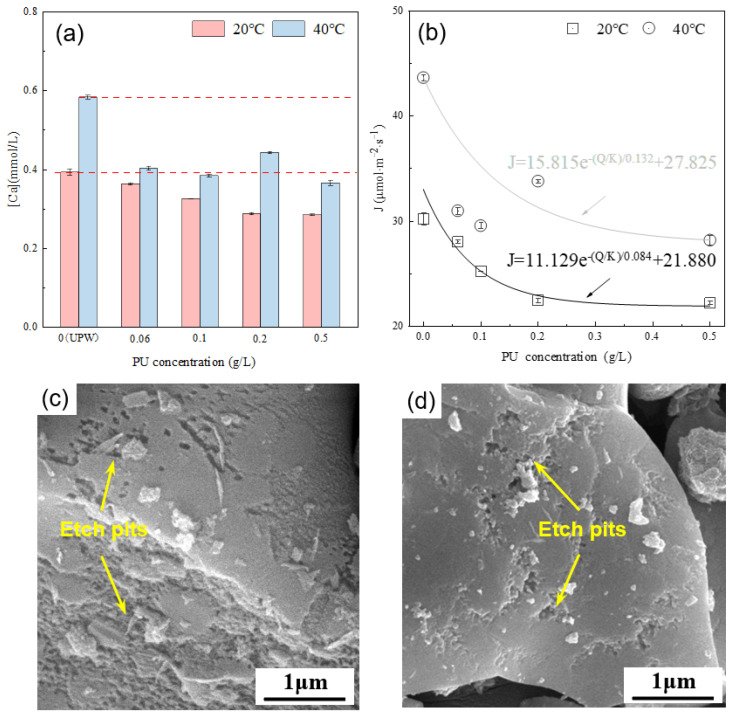
(**a**) Effect of PU concentration on the calcium concentration in the pore solution of an alite suspension after 10 s of dissolution. (**b**) Effect of PU concentration on the dissolution rate of alite powder. SEM image of dissolved alite powders with various PU concentrations at 40 °C: 0.20 g/L PU for (**c**) and 0.50 g/L PU for (**d**). The dissolution time is 60 s.

Figure 9a presents the calcium ion concentration in the alite (90–125 μm) suspension with varying PU concentrations after 10 ± 1 s of dissolution. The results indicate that, compared with dissolution in ultrapure water, the addition of PU consistently reduces the calcium ion concentration in the solution. Calculations of the average dissolution rate of the alite powder reveal an exponential decrease with increasing PU concentration. Moreover, it is evident that at 40 °C, PU solutions of the same concentration exhibit a larger decrease in their calcium ion concentration, indicating a greater inhibitory effect of PU on the dissolution of alite powder at high temperature. From the surface morphology point of view, it is evident that, with PU addition, the surface area of etch pits on the alite surface is significantly decreased compared with that in ultrapure water conditions. At a concentration of 0.5 g/L, most of the alite surface remains smooth after 60 s of dissolution.

Different from COS, PU shows stronger surface electrostatic adsorption, which could block the active site of the alite and inhibit alite particle dissolution. This inhibitory effect increases with higher PU concentrations. Additionally, the reduction in calcium ion concentration may also be attributed to the complexation effect of unabsorbed PU in the solution. The enhanced inhibition of alite dissolution at higher temperature can likely be attributed to the increased adsorption of PU on the alite surface and the stronger ion complexation occurring within the pore solution.

## 4. Conclusions

In this study, we systematically studied the dissolution kinetics of synthetized alite powder by considering the real-time evolution of specific surface area during the alite powder dissolution process. The effect of mixing rate, temperature, saturation level of the pore solution, particle size distribution, and two organic toughening materials, namely COS and PU, on the dissolution of alite powder was investigated. The main conclusions are as follows:

(1) During the dissolution process for alite powder, the specific surface area continuously increases. By conducting BET tests on the alite powder dissolved in ultrapure water at different time intervals, we successfully established a quantitative relationship between the specific surface area of the alite powder and the mass loss during the dissolution process: $\frac{S}{S_{0}}=0.348e^{\left(1-m/m_{0}\right)/0.085}+0.651$.
(2) At 20 °C, the dissolution rate of 45–90 μm alite powder in ultrapure water (UPW) was measured at 33.26 μmol·m^−2^·s^−1^, and for 90–125 μm alite powder, it was 30.22 μmol·m^−2^·s^−1^. At 40 °C, the dissolution rates were 45.33 μmol·m^−2^·s^−1^ and 44.64 μmol·m^−2^·s^−1^ respectively. Temperature has a significant impact on the dissolution rate, exhibiting an approximately 40% increase at 40 °C compared with 20 °C, a pattern similarly observed for stirring speed and solution saturation. In contrast, although particle size does influence the dissolution rate of alite powder, its effect is relatively minor.
(3) The influence of COS on the dissolution rate of alite powder is concentration-dependent. Given the acidic nature of COS, at low concentration (0.02 g/L), it acts as a promoter of dissolution, increasing the dissolution rate of the alite powder. In contrast, at higher concentrations (0.1 g/L and 0.5 g/L), the abundance of COS that covers the particle surface transforms its role into an inhibitor of dissolution, consequently reducing the dissolution rate of the alite powder.
(4) Solutions with varying concentrations of PU consistently lead to a reduction in the dissolution rate of alite powder. This reduction can be attributed to the strong surface electrostatic adsorption of PU, which effectively blocks the active sites on alite particles, thus inhibiting their dissolution. This inhibitory effect becomes notably pronounced at higher temperatures and is likely due to the increased adsorption of PU on the alite surface and the intensified ion complexation occurring within the pore solution.

## Figures and Tables

**Table 1:** Chemical composition of alite powder.

Oxide Composition	CaO	SiO_2_	MgO	Al_2_O_3_	Fe_2_O_3_	TiO_2_
wt. %	69.79	25.93	1.72	1.50	0.84	0.16

**Table 2:** Dissolution rate of alite powder in calcium hydroxide solutions of different concentrations.

[Ca^2+^]/mgL^−1^	Log {Ca^2+^}	log {OH^−^}	Q/K	Dissolution Rate/μmol·m^−2^·s^−1^	
				45–90 μm	90–125 μm
0 (UPW)	/	/	/	33.26	30.22
34.727	−3.167	−2.790	3.256 × 10^−4^	23.38	19.83
247.961	−2.474	−1.989	0.064	13.85	5.37
459.780	−2.287	−1.749	0.298	/	2.62
536.098	−2.245	−1.693	0.425	5.06	/

**Table 3:** Dissolution rate of alite of various particle sizes in water.

System	Polymorph	D [4,3]/μm	SSA/m^2^·g^−1^	T/°C	Dissolution Rate/μmol·m^−2^·s^−1^
45–90 μm	Monoclinic	73.52	0.73	20	33.26
90–125 μm	Monoclinic	108.70	0.41	20	30.22
C_3_S-m [8]	Monoclinic	11.75	0.40	20	74.00
C_3_S-t_1_ [8]	Triclinic	50.16	0.22	25	118.73
	Triclinic				123.30
C_3_S-t_2_ [8]	Triclinic	16.37	0.37	25	103.22
	Triclinic				95.17
	Triclinic				96.52

## Data Availability

The data presented in this study are available on request from the corresponding author. The data are not publicly available due to privacy considerations.
